# Supplementary material for: Whole-body homogenates restore disrupted microbiota composition in a model insect better than feces or no restoration treatment
Source: bioRxiv. 2025 Nov 17:2025.11.17.688872. Preprint. [Version 1] doi: 10.1101/2025.11.17.688872 (PMC12667818; doi:10.1101/2025.11.17.688872)
Supplement: Supplement 1 [file NIHPP2025.11.17.688872v1-supplement-1.pdf]

bioRxiv preprint doi: https://doi.org/10.1101/2025.11.17.688872; this version posted November 17, 2025. The copyright holder for this preprint (which was not certified by peer review) is the author/funder, who has granted bioRxiv a license to display the preprint in perpetuity. It is made available under aCC-BY 4.0 International license.

SUPPORTING INFO

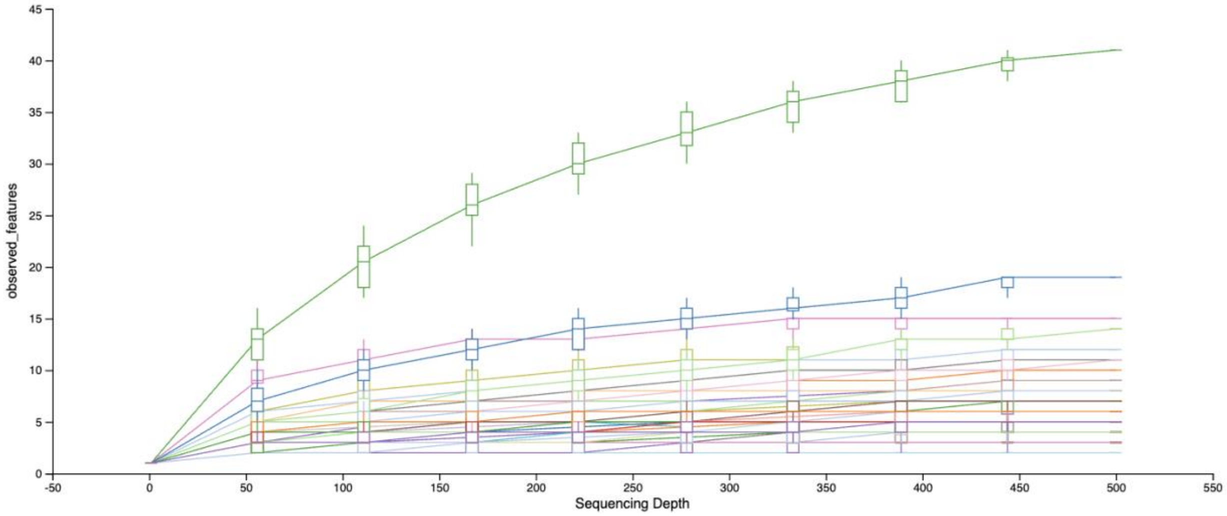

Figure S1. Rarefaction curve showing saturating sampling levels were approached. The curves show observed feature counts (y-axis) as a function of sequencing depth (x-axis). Most samples reached saturation by 500 sequences, supporting the chosen rarefaction depth for downstream diversity analysis.

bioRxiv preprint doi: https://doi.org/10.1101/2025.11.17.688872; this version posted November 17, 2025. The copyright holder for this preprint (which was not certified by peer review) is the author/funder, who has granted bioRxiv a license to display the preprint in perpetuity. It is made available under aCC-BY 4.0 International license.

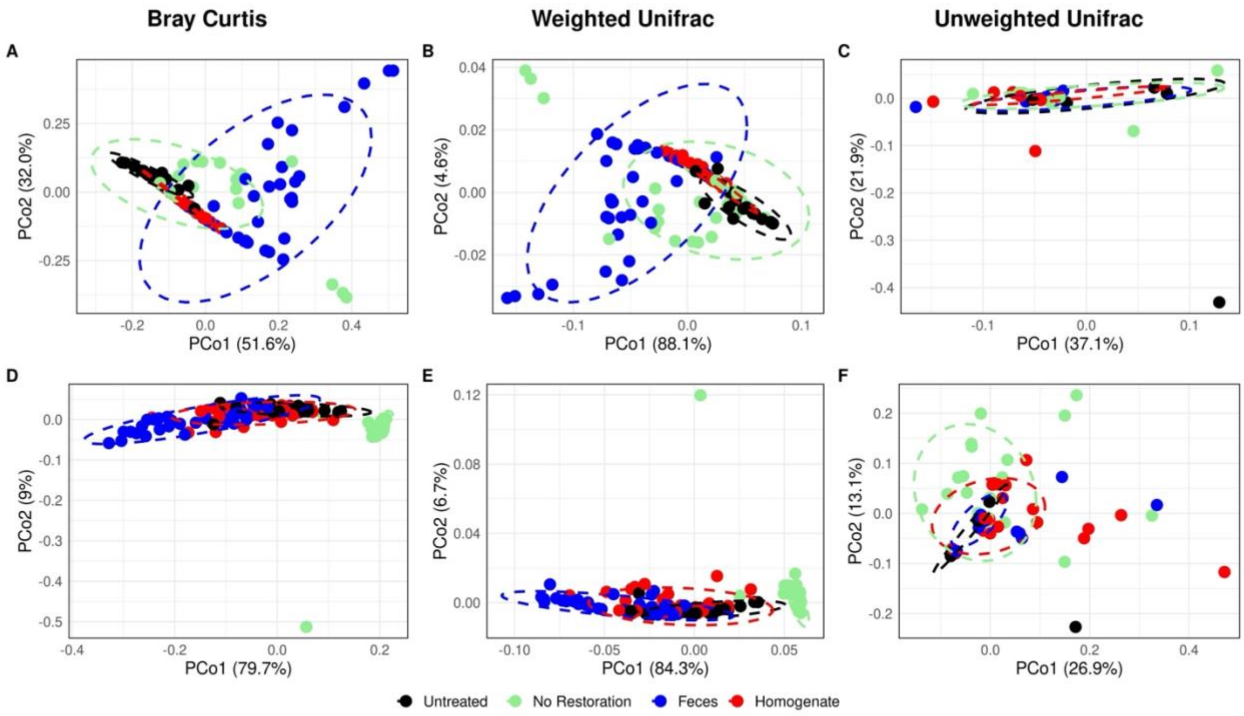

Figure S2. Principal coordinates analysis (PCoA) of W+ and W- flies. Principal Coordinates ordinations for A-C) W+ and Untreated, D-F) W- and Untreated flies.

bioRxiv preprint doi: https://doi.org/10.1101/2025.11.17.688872; this version posted November 17, 2025. The copyright holder for this preprint (which was not certified by peer review) is the author/funder, who has granted bioRxiv a license to display the preprint in perpetuity. It is made available under aCC-BY 4.0 International license.

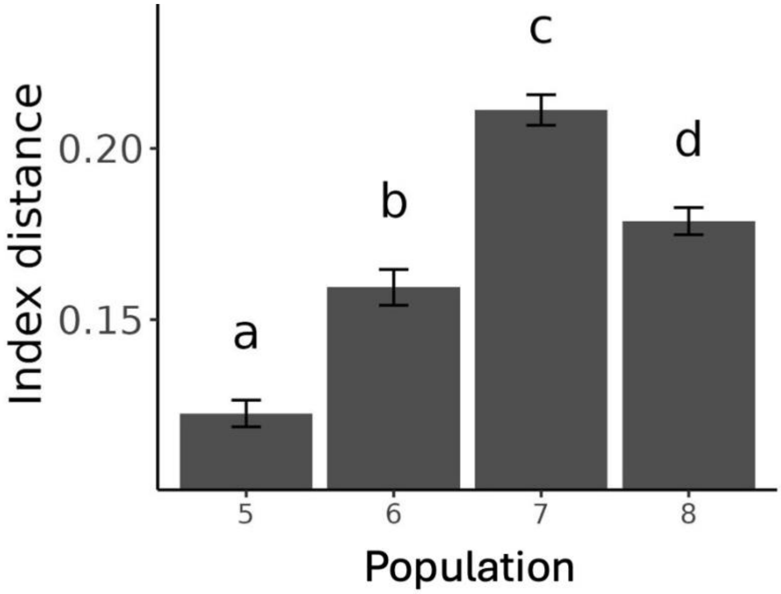

Figure S3. Bray-Curtis distances between W- Homogenate-treatment populations and Untreated flies. Compact letter displays show significant differences in the beta-diversity distances of W+ Populations 5-8 relative to Untreated flies. Population 5 was used in Figure 5 assays.
